# Integrating Gestures and Words to Communicate in Full-Term and Low-Risk Preterm Late Talkers

**DOI:** 10.3390/ijerph19073918

**Published:** 2022-03-25

**Authors:** Chiara Suttora, Annalisa Guarini, Mariagrazia Zuccarini, Arianna Aceti, Luigi Corvaglia, Alessandra Sansavini

**Affiliations:** 1 Department of Psychology “Renzo Canestrari”, University of Bologna, 40126 Bologna, Italy; annalisa.guarini@unibo.it; 2 Department of Educational Sciences, University of Bologna, Via Filippo Re 6, 40126 Bologna, Italy; mariagrazia.zuccarini@unibo.it; 3 Neonatal Intensive Care Unit, IRCCS Azienda Ospedaliero-Universitaria Bologna, Via Massarenti 9, 40138 Bologna, Italy; arianna.aceti2@unibo.it (A.A.); luigi.corvaglia@unibo.it (L.C.); 4 Department of Medical and Surgical Sciences, University of Bologna, Via Massarenti 9, 40138 Bologna, Italy

**Keywords:** pointing, representational gestures, gesture–word combinations, late talkers, language delay, low-risk preterm birth, risk and protective factors

## Abstract

Young children use gestures to practice communicative functions that foster their receptive and expressive linguistic skills. Studies investigating the use of gestures by late talkers are limited. This study aimed to investigate the use of gestures and gesture–word combinations and their associations with word comprehension and word and sentence production in late talkers. A further purpose was to examine whether a set of individual and environmental factors accounted for interindividual differences in late talkers’ gesture and gesture–word production. Sixty-one late talkers, including 35 full-term and 26 low-risk preterm children, participated in the study. Parents filled out the Italian short forms of the MacArthur–Bates Communicative Development Inventories (MB–CDI), “Gesture and Words” and “Words and Sentences” when their children were 30-months-old, and they were then invited to participate in a book-sharing session with their child. Children’s gestures and words produced during the book-sharing session were transcribed and coded into CHAT of CHILDES and analyzed with CLAN. Types of spontaneous gestures (pointing and representational gestures) and gesture–word combinations (complementary, equivalent, and supplementary) were coded. Measures of word tokens and MLU were also computed. Correlational analyses documented that children’s use of gesture–word combinations, particularly complementary and supplementary forms, in the book-sharing session was positively associated with linguistic skills both observed during the session (word tokens and MLU) and reported by parents (word comprehension, word production, and sentence production at the MB–CDI). Concerning individual factors, male gender was negatively associated with gesture and gesture–word use, as well as with MB–CDI action/gesture production. In contrast, having a low-risk preterm condition and being later-born were positively associated with the use of gestures and pointing gestures, and having a family history of language and/or learning disorders was positively associated with the use of representational gestures. Furthermore, a low-risk preterm status and a higher cognitive score were positively associated with gesture–word combinations, particularly complementary and supplementary types. With regard to environmental factors, older parental age was negatively associated with late talkers’ use of gestures and pointing gestures. Interindividual differences in late talkers’ gesture and gesture–word production were thus related to several intertwined individual and environmental factors. Among late talkers, use of gestures and gesture–word combinations represents a point of strength promoting receptive and expressive language acquisition.

## 1. Introduction

### 1.1. Gestural Communication in Typical Development

Before children become able to utter their first words to refer to people or objects in their proximal context, they start communicating through other expressive means, especially gestures. First emerging gestures, typically defined as deictic, are used by children with the intention to convey requests to communicative partners or to direct their attention to objects and events of interest. These functions were respectively addressed as proto-imperative and proto-declarative functions by Bates and colleagues [1].

Among deictic gestures, pointing—i.e., the extension of the index finger toward a specific object or event [2]—is certainly the most relevant, as an extensive amount of literature has shown. The use of pointing expands children’s experiences and perspectives: by the use of pointing as a joint attention behavior, they can share their interests with other people, spending more time on social and verbal exchanges that are fundamental for language acquisition. In this direction, concurrent and predictive associations between pointing onset and use and children’s linguistic competencies are well documented in the literature for children with typical [3,4] and atypical development [5,6,7,8,9].

After the emergence of deictic gestures, at approximately children’s first birthday, other types of gestures appear in their communicative repertoires. Unlike early forms of gesturing, these brand-new gestures are symbolic as their referential meaning is independent of their context of use [10]. These gestures have been defined as symbolic, representational, or referential, and they can be distinguished into two sub-categories: (a) conventional gestures culturally defined (e.g., waving goodbye); and (b) iconic gestures that convey actions or attributes of the represented object or event (e.g., moving arms in the air to indicate a bird). With representational gestures (throughout the manuscript we are going to use the term “representational” to refer to this class of gestures), children begin to express symbolic meanings in a modality, the gestural, which is easier to master than the verbal, once the required cognitive competences are in place. Representational gestures are also associated with children’s language development, with data showing that the use of such gestures correlates with the acquisition of receptive and expressive lexicon [4], social lexicon [11], and the onset of verbs six months later [12].

A further stepping stone in the development of gestural communication is represented by the emergence of gesture–word combinations that allow children to express two pieces of information within the same utterance before the onset of two-word combinations. Literature indicated that gesture–word combinations “pave the way” for language development, predicting the onset of two-word combinations and the development of morphosyntactic skills, in terms of mean length of utterances (MLU) [9,13,14,15].

### 1.2. Gestural Communication in Children with Language Delay

Between the second and the third years of life, some children—with a prevalence comprised between 9% and 21%—show a delay in language development, as documented by studies conducted in Europe, Canada, Australia, and the US [16,17,18,19,20,21]. These children are identified as late talkers. Between the ages of 18 and 35 months, they exhibit an expressive vocabulary at or below the 10th percentile with respect to standardized tools, as the MacArthur–Bates Communicative Development Inventory (MB–CDI) [22] or the Language Development Survey (LDS) [23], in the absence of neurological, sensory, cognitive, or socioemotional deficits. About two-thirds of late talkers, commonly referred to as late bloomers, will recover from this initial delay showing a catch-up in their linguistic developmental path after their third birthday [24]. About one third of late talkers, however, will show persistent difficulties in their linguistic abilities with cascading effects on later academic skills. Besides their limited expressive lexicon, late talkers also often exhibit weaknesses in other linguistic aspects, such as receptive vocabulary [25,26] and phonological and morphosyntactic skills [20,27].

Beyond the linguistic area, seminal works on late talkers focused their investigation on the association between language and cognition, specifically emphasizing the relationship between language and gestures. Research from Thal and collaborators [28,29,30,31] began to shed light on such a link, trying to comprehend whether late talkers’ use of gestures was different from that exhibited by typically developing children. These works examined elicited and spontaneous gesture production in a small sample of late talkers from 18 to 28 months, further observed at a one-year follow-up. The primary hypothesis was that late talkers would have produced more communicative gestures to overcome their linguistic difficulties in the absence of cognitive fragilities. Their results showed that children with language delays did not exhibit a greater production of gestures than age-matched controls. However, by dividing their sample according to the results of their follow-up, Thal and colleagues noted late bloomers appeared to exhibit a greater use of communicative gestures, both deictic and representational, than truly delayed late talkers. In summary, children recovering from the early delay showed a compensatory use of communicative gestures, whereas children who kept on having language difficulties did not.

Apart from these early works, literature regarding late talkers’ communicative gesture production is still limited, although there has been a renewed interest in this topic in the last decade. O’Neill et al. [32,33], following the studies of Thal et al. [29,30], investigated the use of communicative gestures in children with expressive delay and children with receptive/expressive delay and how this behavior was predictive of their later outcomes. At 2–3 years of age, compared with children with expressive delay only, children with both receptive and expressive delay showed a lower amount of communicative gestures—as assessed with the Communication and Symbolic Behavior Scales [34]—and also difficulties in their level of symbolic comprehension. For all children, gesture production was related to receptive but not expressive language. Furthermore, gesture use at 2–3 years of age was predictive of children’s expressive linguistic competencies at a follow-up when children were 4–5 years old [33], a result corroborating the first findings of Thal et al. [29]. Recently, Lüke et al. [5] also highlighted the strong predictive role of gesture—particularly pointing—in the development of language delay. In their sample, children who did not exhibit finger pointing at 12 months were most likely to become late talkers at 2 years. Consistent with Lüke et al. [5], Sansavini et al. [8] reported that siblings with no diagnosis of autism spectrum disorder and preterm children with extremely low gestational age, who developed a language delay between the second and third year of life, compared to those who did not develop a language delay, showed an absence or lower rates of pointing at 18 months. Again, similar findings were achieved by Manwaring et al. [6]. They observed a scarce use of deictic and conventional gestures, assessed with the Communication and Symbolic Behavior Scales and during a naturalistic parent–child interaction, in children with language delay. In summary, most of the reviewed literature focused on the role of gestures, primarily pointing, in predicting the emergence of language delay in children belonging to the general population or to populations with risk conditions such as familiarity for language delay, familiarity for autism spectrum disorder, or preterm birth. Despite these relevant contributions, the gestural communicative repertoires of children with language delay have not been extensively described, especially after the age of 2 years, when the presence of a language delay is established. In addition, the literature reported only limited data regarding the onset and the use of gesture–word combinations in this population [35], which is a relevant predictor of children’s access to word combinations [13,14,15].

### 1.3. Individual and Environmental Factors of Risk for Language Delay

Several individual and environmental factors have been pointed out as potential predictors of language delay in children. Among the earlier, being male, having a family history of language or learning delay/disorder, or having neonatal risk conditions, such as being born preterm or with a low birthweight, represent significant risk factors for the emergence of an expressive language delay. As for the latter, economic, social, and educational family background—generally expressed by low socioeconomic status (SES) and/or low parental education—are relevant contributors to language delay [16,17,36,37,38,39,40,41,42]. Children in poverty have fewer familiar resources and limited educational options than their peers living in middle and high SES contexts; these limited options can lead to fewer opportunities of interaction and the exposition to inadequate linguistic environments [43,44,45]. At the same time, lower parental educational levels, which are often intertwined with economic and/or educational poverty, are associated with children being exposed to a lower amount and quality of talking, contributing to creating a less adequate environment for children’s language development [43,46].

Besides the factors mentioned above, other variables caught the attention of researchers and clinicians who were trying to explain variability in children’s early communicative and language development. Birth order is often considered a factor for the prediction of language development, with first-borns being depicted as having a slight advantage with respect to later-born children, although findings are mixed. On the one hand, first-born children demonstrate stronger lexical and grammatical development than later-born children [17,47,48]. The first-born benefit seems to be related to the amount and the quality of time that first-time parents can dedicate to their children which, by contrast, is more limited when a household is composed of more than one child. On the other hand, other studies found that later-born children exhibit stronger social language skills, probably as a result of having older siblings playing the part of socialization agents in their interactive and communicative development [49,50,51,52].

Another variable that some studies have considered is maternal age. Literature focusing on the quality of caregiver–infant interaction [53,54,55] documented how early and late parenthood can represent a risk factor for maternal responsivity and sensitivity. Some studies revealed that very young and older maternal ages can be associated with a developmental language disorder, but findings are unclear about this association with the emergence of language delay before 3 years of age [17,56,57,58,59]. Finally, scholars have taken the relationship between children’s cognitive and linguistic abilities into account. Sansavini et al. [20] reported that children described as having a poor or a weak profile in their language development at 30 months showed lower cognitive scores than children with an average linguistic profile. Similarly, Desmarais et al. [60], exploring the linguistic skills of a group of late talker children, identified a cluster of children with the weakest language abilities who also exhibited weak cognitive skills.

### 1.4. Aims of the Study

The present study aimed to investigate late talkers’ use of gestures, gesture–word combinations and their associations with word comprehension and word and sentence production. Based on previous literature, we expected a frequent use of gestures in late talkers, compensating for their lexical delay. At the same time, we hypothesized significant associations between children’s use of gesture and word comprehension, and between children’s use of gesture–word combination and word and sentence production. In typically developing children, the use of word–gesture combination is indeed considered a bridge to word–word combination [14]. A further aim of the study was to examine which individual and environmental variables, among those known to be related to language development, predicted late talkers’ use of gestures and gesture–word combinations. We expected both individual and environmental factors, such as male gender, birth condition, birth order, family history of language and/or learning disorders, and parental age and education, to play a role for gesture and gesture–word combination development similar to that shown for language development [4,16,17,61].

## 2. Material and Methods

### 2.1. Participants

Participants were recruited within a screening project (for details about the screening, see Sansavini et al., 2021 [20]) conducted on language development of children born at the Sant’Orsola-Malpighi Hospital of the University of Bologna. The screening involved 200 participants, 100 born preterm and 100 full-term, that were recruited according to the following criteria: (a) being monolingual or mainly exposed (>65% of daily exposure) to the Italian language from birth onward; (b) being born full-term (i.e., with a gestational age ≥ 37 weeks) or low-risk preterm (i.e., with a gestational age < 37 weeks and lack of severe perinatal complications); and (c) not having a major cerebral damage and/or congenital malformations, visual, hearing, or motor impairments, or severe cognitive deficits (identified by a Bayley-III composite cognitive score < 70). Out of the 200 participants screened, 61 children identified as late talkers with the procedure specified in Section 2.3 participated in the present study.

Table 1 includes the biological, medical, and socio-demographic characteristics of late talker children and their parents. Out of 61 late talkers, 35 (57.4%) were born full-term and 26 (42.6%) low-risk preterm (see Appendix A Table A1 for the biological, medical, and socio-demographic characteristics of full-term and low-risk preterm children, respectively). The children’s Bayley composite cognitive scores are also reported in Table 1 and Appendix A (the test is described in the Tools paragraph). Socio-demographic characteristics were obtained by administering an ad-hoc questionnaire to parents, whereas biological and medical information were retrieved from the infants’ birth and medical history database of Sant’Orsola-Malpighi Hospital of the University of Bologna.

### 2.2. Tools

#### 2.2.1. MacArthur–Bates Communicative Development Inventories

The short forms of the Italian versions of the “Gesture and Words” and “Words and Sentences” MB–CDI [62] were employed. The first part of the “Gestures and Words” form, consisting of a 100-word list, was used to assess each child’s word comprehension. Parents were asked to check the words their child understood; a score of 1 was given for each item checked. Word comprehension (i.e., the total number of words understood) was computed. The second part, including a list of 18 gesture/action production items, was used to assess each child’s use of gestures and symbolic actions. Parents were asked to check the gestures/actions their child produced; a score of 1 was given for each item checked. Gesture/action production (i.e., the total number of gestures/actions produced) was computed. Typically, the “Gestures and Words” form is used with children aged 8 to 24 months, but as late talkers could present with receptive and gestural delays, this form was administered to assess these competences, as done in a previous study on 30-month-old children [20]. The first part of the “Words and Sentences” form, including a list of 100 words, was used to assess word production. Parents were asked to indicate if their child spontaneously produced each word. A score of 1 was given for each item checked. Word production (i.e., the total number of words produced) was computed. The second part, including 12 pairs of sentences that each consisted of an incomplete and a complete sentence, was used to assess sentence production. For each pair of sentences, parents were asked to check the sentence that best represented their child’s sentence production. A score of 1 was given for each item checked. Sentence production (i.e., the total number of sentences produced) was computed.

#### 2.2.2. Bayley Scales of Infant and Toddler Development

The Bayley Scales of Infant and Toddler Development, Third Edition (BSID-III) [63,64] was used to assess children’s cognitive level and to ascertain that children had a composite cognitive score ≥ 70. The Bayley-III Scales are a valid and widely used tool for research and for clinical practice with satisfactory reliability and validity values [63].

### 2.3. Procedure

Parents were asked to fill out the short forms of the Italian versions of the MacArthur–Bates Communicative Development Inventories (MB–CDI), “Gesture and Words” and “Words and Sentences” [62]. Children were identified as late talkers if their expressive vocabulary fell at or below the 10th percentile with respect to the data of the Italian population [65]. After the screening, when children were near the age of 31 months (*M* = 31.74, *SD* = 1.51), they were invited to the Developmental Psychology Lab at the University of Bologna to assess their cognitive and linguistic skills directly. For preterm children, age was corrected (i.e., calculated from the expected date of birth, assuming 40 weeks of gestation) in order to consider their level of neurobiological maturation as done in previous studies [38]. At the assessment, full-term children’s mean chronological age was 31.01 months (*SD* = 1.13), low-risk preterm children’s mean corrected age was 31.28 months (*SD* = 0.99) with a mean chronological age of 32.71 months (*SD* = 1.13). There was no significant difference between preterm children’s corrected age and the chronological age of full-term children, *t* (59) = −0.87, *p* = 0.38. To exclude any cognitive delay, children’s cognitive skills were assessed through the Bayley Scales of Infant and Toddler Development, Third Edition (BSID-III) [63].

Children were also invited to a book-sharing reading session with one of their parents (usually the mother, except for three full-term children who were observed with their fathers). The session was video-recorded and lasted approximately 10 min (*M* = 9.78, *SD* = 1.61). The dyads were free to interact by sharing two books (“L’elefante pittore” [The elephant painter] and “Anna va alla scuola materna” [Anna goes to kindergarden]) selected according to participants’ age and linguistic skills.

The study was approved by the Bologna Health Authority’s Independent Ethics Committee (approval numbers: EM 194-2017_ and EM 193-2018_76/2013/U/Sper/AOUBo). All parents of eligible children were informed about the investigation and asked to fill in the informed written consent for participation in the study, data analysis, and data publication.

### 2.4. Coding and Measures

#### Coding of Children’s Spontaneous Gestural and Verbal Production

Children’s spontaneous communicative gesture and word production were observed during a parent–child shared book reading session. They were transcribed and coded into the CHAT format of the Child Language Data Exchange System (CHILDES) and analyzed with CLAN software [66]. A gesture was defined as communicative if it was used with the aim of getting the partner’s attention or interest [67]. Gestures were classified into the following categories [9,12,68,69,70]:

(a) Pointing gesture: clear extension of the index finger toward a proximal or distal object, picture, person, or event for the purpose of identifying a referent in a given context and sharing attention on it with the partner (declarative function). Other deictic gestures (i.e., showing, giving, and requesting/reaching) were not coded as their production was limited in a book-sharing context and, in the case of requesting/reaching, the declarative function was lacking.
(b) Representational gestures: this category includes both conventional and iconic gestures. Conventional gestures refer to culturally-based gestures produced and understood by all members of a given cultural group (e.g., waving goodbye, nodding, etc.). Iconic gestures represent the form or function of an object, an action, or an event (e.g., waving the arms to indicate a bird or a plane).

The observer further noted whether gestures were co-temporally accompanied by word production. Words and gestures that were produced simultaneously were classified as gesture–word combinations and were further divided into the following categories [13,14,15,71]: (a) complementary combinations in which the gesture identifies the referent and the word labels it (e.g., the child points at a picture of a dog in the book saying “doggy”; the child points at an image in the book saying “look”); (b) equivalent combinations in which the gesture and the word convey the same referent (e.g., the child nods and says “yes”; the child pretends to wash his hands and says “wash”); and (c) supplementary combinations in which the gesture and the word convey two different referents (e.g., the child nods and says “ball”; the child shakes his head no and says “small”).

As regards children’s lexical productivity, the number of word tokens (including nouns, verbs, adjectives, function words, and yes/no tokens) were computed after excluding children’s interjections and repetitions of parents’ speech. To assess children’s grammatical complexity, the mean length of utterances (MLU) was calculated based on the utterances produced during the book-sharing session; for four full-term children not showing any word production, MLU could not be computed.

The frequencies per 10 min of total gestures, pointing gestures, representational gestures, gesture–word combinations and their types (complementary, equivalent, and supplementary), and word tokens were computed for each participant.

### 2.5. Reliability

A second independent observer (i.e., the first author of this paper) coded 29% of the sessions to assess interobserver reliability. Regarding the coding of communicative gestures, the percentage of agreement between observers was 85%. The observers’ agreement on pointing and representational gestures corresponded to a Cohen’s Kappa of 0.98; on types of gesture–word combinations (complementary, equivalent, and supplementary) to a Cohen’s Kappa of 0.86. On the whole, reliability resulted high. With regard to children’s linguistic outcomes in terms of the frequency of word tokens and MLU, interrater agreement was achieved by calculating the intraclass correlation coefficients (ICC), with ICCs > 0.98.

### 2.6. Statistical Analyses

Before addressing our aims, we checked the distribution of the study’s variables for normality. As Kolmogorov–Smirnov and Shapiro–Wilk tests indicated that most variables were not normally distributed (*p* < 0.01), a rank transformation was applied to overcome this issue. Data analyses were performed with IBM SPSS Statistics 27 (Armonk, NY, USA), using a bilateral test with *p* set at <0.05.

For the first aim, the mean and standard deviation of variables were reported to describe late talkers’ gestures, gesture–word combinations, word tokens, and MLU during the book-sharing session. In addition, descriptive data concerning late talkers’ gesture/action production and word comprehension reported at the MB–CDI “Gestures and Words” short form, and word production and sentence production reported at the MB–CDI “Words and Sentences” short form were reported. Correlational analyses were carried out among measures regarding children’s use of gestures and gesture–word combinations (i.e., children’s communicative gestures, gesture–word combinations and their types, and MB–CDI gesture/action production) and lexical and grammatical measures as observed during the book-sharing session and reported at the MB–CDI (i.e., word tokens, MLU, word comprehension, word production, and sentence production).

The second set of correlations was conducted to preliminarily assess the associations between children’s gestural measures and a group of individual and environmental factors that were hypothesized to predict children’s use of gestures and gesture–word combinations. In these analyses birth status, gender, family history of language and/or learning disorders, birth order, parental age and education, and cognitive score were included as predictors. Children’s chronological age was also included in order to control for it. Individual and environmental variables showing significant associations with measures of children’s gesture and gesture–word combination were then considered in a set of stepwise linear regressions.

## 3. Results

### 3.1. Descriptive Analyses

Descriptive analyses reported in Table 2 indicated that children, during the 10-min book-sharing session, produced a mean of 36.51 communicative gestures with a greater amount of pointing gestures with respect to representational gestures. Mean gesture–word combinations were 7.29 in the 10-min session; complementary combinations were the most common followed by a small amount of equivalent and supplementary combinations. Children produced on average almost 22.68 word tokens, showing a mean MLU of 1.09 words.

Descriptive statistics for children’s scores obtained with the administration of the MB–CDI Gestures and Words Short Form and the Words and Sentences Short Form are summarized in Table 3. Children produced a mean of 15.18 actions/gestures over a total of 18 items, and they exhibited a mean of 87.57 in word comprehension and 18.49 in word production over a total of 100 items. Regarding sentence production, children showed a mean of 2.84 sentences.

### 3.2. Relationship between Gesture Use and Lexical and Grammatical Abilities in Children with Language Delay

Table 4 reports the intercorrelation coefficients between the study’s main variables. The results showed that total gesture–word combinations, as well as complementary and supplementary combinations, were positively associated with children’s lexical and grammatical abilities—in particular with word tokens and MLU produced during the 10-min book-sharing session, and word production and sentence production reported at the MB–CDI. Equivalent combinations were positively associated with word tokens. Supplementary combinations were also positively associated with word comprehension reported at the MB–CDI. Finally, gesture/action production reported at the MB–CDI was positively associated with word comprehension reported at the MB–CDI.

### 3.3. Individual and Environmental Factors Predicting Variability in the Use of Gestures and Gesture–Word Combinations

The results of preliminary correlations aimed at investigating the associations between measures of late talkers’ gestures and gesture–word combinations and the individual and environmental variables supposed to impact such abilities are reported in Appendix A Table A2. As maternal and paternal education did not show significant correlations with any communicative behavior, they were excluded as possible predictors from regression analyses. With regard to maternal and paternal age, as both variables were similarly associated with children’s use of gestures and pointing gestures, the mean of these two variables was included in the regression models (i.e., parental age).

Table 5 reports the results of multiple regressions performed to assess the contribution of individual and environmental factors in explaining children’s variability in the use of gestures and gesture–word combinations. Birth status, gender, birth order, family history of language and learning delays, cognitive score, and chronological age were included as individual factors; mean parental age was considered as an environmental factor in the models. Results showed that total gestures were positively predicted by being low-risk preterm and later-born, whereas negatively predicted by being male and having older parents. Similarly, pointing gestures were positively predicted by being low-risk preterm and later-born, whereas negatively predicted by having older parents. Representational gestures were positively predicted by a family history of language and learning disorders. Gesture–word combinations were positively predicted by being low-risk preterm. Specifically, complementary combinations, were positively predicted by being low-risk preterm, whereas negatively predicted by male gender. Supplementary combinations were positively predicted by being low-risk preterm and having a higher cognitive score. Finally, gesture/action production, as assessed by the MB–CDI, was negatively predicted by male gender.

## 4. Discussion

The literature widely acknowledges that the development of gestural communication supports language development. Indeed, gestures allow children to share interests with others, express meanings for which they do not possess a verbal label, and—when combined with words—begin to combine meanings within an utterance. Although the importance of early gestural communication has been highlighted by scholars worldwide and explored in different populations of children with typical and atypical development, research concerning children with language delay is still scant and mixed in its findings. The primary goal of the present study was to investigate the use of gestures and of gesture–word combinations and their associations with word comprehension and word and sentence production in a sample of children identified as late talkers at the age of 30 months. Gestures and children’s linguistic abilities were examined via a parental questionnaire and by observing them during a parent–child book-sharing session. A further aim was to examine which individual and environmental variables, among those known to be related to language delay, predicted late talkers’ use of gestures and gesture–word combinations.

Regarding the first aim, we observed that children exhibited a fair use of communicative gestures during the sessions, with a prevalence of pointing gestures—representing approximately 80% of the gestures produced—and a more limited use of representational gestures. The high percentage of pointing gestures is likely to be due to the specificity of the observational context, as book-sharing is likely to elicit use of pointing by both children and parents [72]. This result is in line with the finding retrieved by Lavelli et al. [73] that documented a higher use of deictic over representational gestures in children with expressive specific language impairment and two control groups of age-matched and language-matched typically developing children during a shared book reading with their mothers.

In our sample, almost 20% of communicative gestures were produced in combination with a verbal element. In children with typical and atypical development, the use of gesture–word combinations favored the transition to word–word combination, as by putting together two elements in a single communicative act, children begin to master the combination of multiple meanings [13,14,15]. In children with language delay, the use of gesture–word combinations has been very scarcely investigated. Fasolo & D’Odorico [35] considered this type of production in late talkers’ communicative repertories but without differentiating between gestures produced with a preverbal or a verbal element. Our study represents a new attempt to address more in-depth the issue of gesture–word combinations in children with language delay. The results indicated that children mostly exhibited complementary combinations, with equivalent and supplementary forms being more limited in their amount. This outcome is explained by children’s greater use of pointing gestures, rather than those that are representational, as complementary combinations always include a deictic element, and it is similar to that observed in typical development [67].

Concerning the associations between gestural and linguistic measures, our data showed mixed findings based on the type of gesture assessment, either as communicative gestures observed during book-sharing or as reported by parents at the MB–CDI. Our findings revealed that when we consider all gestures—including pointing and representational gestures separately—observed during book-sharing, they did not correlate with children’s word comprehension or with their word and sentence production measures as reported by parents at the MB–CDI. Conversely, gestures reported by parents at the MB–CDI were positively related to children’s word comprehension reported with the same parental questionnaire, even if this correlation result was weak when compared to the other correlational results. Looking at the existing literature, there is not much consistency in the findings; O’Neill & Chait [32] found that among 2–3 year-old late talkers, the use of gestures elicited in a structured task correlated with the receptive but not the expressive language score. Our mixed results may be explained by differences between observation in a book-sharing contest and the MB–CDI parental report in capturing gesture ability. Through observation of book-sharing interactions, pointing and representational gestures were captured in their overall frequency of use (gesture tokens) in a specific context (book-sharing). By contrast, the gestural data collected at the MB–CDI inform us whether children are able to use gestures and symbolic actions to share attention and communicate meanings with their partners across different contexts, giving a more global measure of gesture/action production, but not specified in frequency. Another possible explanation of the present findings is that correlations among MB–CDI gesture/action production and word comprehension are significant, even if weak, as both measures were reported by children’s parents.

Looking, however, specifically at the associations between gesture–word combinations and children’s linguistic abilities, findings indicated that total gesture–word combinations, as well as complementary, equivalent, and supplementary combinations, were positively related to children’s word and sentence production as observed during the book-sharing session and with the MB–CDI questionnaire. Supplementary gesture–word combinations were also positively associated to children’s word comprehension. These results extend to late talkers and they are evidence of the strict relationship between word comprehension and gesture/action and word production, as found in children’s typical development between the first and second years of life [4,74]. Longitudinal studies addressing populations with typical and atypical development, such as children born preterm and children with Down syndrome, found gesture–word combinations to be predictive of later children’s advances in their lexical and grammatical abilities [9,13,14,71,75]. Our data offer a cross-sectional perspective indicating that gesture–word combinations are concurrently associated with late talkers’ receptive and expressive linguistic abilities. Further longitudinal studies could verify the predictive role of gesture–word combinations on later language skills in late talker children.

The last aim of the study was to identify individual and environmental variables that account for interindividual differences in gesture and gesture–word use. In the first place, we found a male disadvantage in using gestures—as observed in the book-sharing session and with the parental report—and complementary combinations, an outcome that seems to reflect the more general effect of male gender on language development [17,56]. Literature indicates that the risk of developing a language delay can be three-times higher for boys than girls [17]. As for communicative gesture development, similar outcomes were found by Sansavini et al. [4] for children with typical development. Similar to a previous study [22] documenting an advance for girls in the acquisition of gesture/actions, as measured with the MB–CDI, Sansavini et al. [4] found girls had an advantage in the production of gestures and object-actions separately considered up to the second year of life. An advantage of girls over boys on gesture use has also been documented in typically developing children aged 14 to 34 months by Özçalişkan and Goldin-Meadow [61]; specifically, boys were 3 months delayed compared to girls in the onset of supplementary gesture–word combination. Based on previous literature, these authors suggested that the risk for gesture use associated with male gender can be linked to sex differences in motor development, as literature documented an advantage for girls in fine-motor abilities. Their hypothesis supports the idea that biological factors are primary contributors to sex differences in children’s use of gesture and later language development. Other scholars, however, without belittling the role of biological factors, support a biopsychosocial perspective claiming that an initial biological female advantage can interact with environmental aspects—such as the way parents respond to children’s gestures and words—creating a positive reinforcing cycle that can explain sex differences in language skills [76,77]. Our study allows us to generalize this finding to children with language delay, confirming male gender as a risk factor for the development of action/gestures, gestures and gesture–word combinations up to the third year of life.

Another variable that affected children’s use of communicative gestures was birth order, as later-born children showed greater use of gestures and in particular of deictic gestures than first-born children. Regarding the development of communicative gestures, we found only one study addressing the effect of birth order in 18-month old children with typical development, with results depicting a lack of differences between first- and later-born children [48]. As for the broader development of children’s linguistic and communicative abilities, as previously highlighted, findings documenting birth order effects are mixed. A consistent number of studies documented a first-born’s advantage in lexical and grammatical skills. Other studies pointed at a later-born’s advantage in social language abilities [17,47,49,52]. Concerning the latter, Pine [78] found that at the 100-word stage second-born children had a higher percentage of frozen phases and of deictic personal pronouns, such as “me” or “mine”. Our findings documenting an advantage in the production of gestures and deictic gestures in later-born late talkers can be read as a slight benefit for these children in the development of socio-communicative skills. However, with such limited evidence on this topic—together with the intervening effects of other factors, such as parental SES [79]—it is difficult to draw any conclusion about the effect of parity on late talkers’ gesture development.

Our findings also revealed that preterm birth, in our case low-risk preterm birth, contributed in determining individual differences in the use of gestures, pointing gestures, and gesture–word combinations, including complementary and supplementary forms, with low-risk preterm birth predicting more favorable outcomes than full-term birth in late talker children. Although this finding can be considered as unexpected, it should first be noted that preterm children in our sample did not present severe risks or medical conditions at birth, being born with a low degree of immaturity. Furthermore, literature addressing preterm children’s development and communicative gestures—mostly conducted on very preterm samples—documented mixed results. Some studies reported the presence of delay or difficulties in using communicative gestures [38,80] whereas others identified performances similar to full-term children [9,81]. Examining gesture production in a sample of very preterm infants at 12 and 18 months with the Gesture and Word form of the Italian MB–CDI, Sansavini et al. [38] found that very preterm children showed a slower acquisition in gesture/action production with difficulties becoming more evident toward 18 months, with a greater effect in the production of gestural actions (functional, pretending, imitating). By contrast, Cattani et al. [81], assessing a sample of preterm children rather heterogeneous for their level of immaturity at birth, found corrected-age appropriated performances in gesture and action use. Regarding gesture–word combinations, however, both Sansavini et al. [68] and Suttora & Salerni [9] found that preterm children were less able to convey meanings via the bimodal gesture–speech modality at two years of age. It should be observed that the children involved in the abovementioned studies were extremely and very preterm children, respectively, populations considerably at higher risk than those involved in the present study. A second major element to consider to better comprehend the role of preterm birth in determining differences in the use of gestures and gesture–word combinations is the use of corrected age for preterm participants. This practice is widely suggested by literature but its use has been at the center of scholars’ discussions [81,82]. Cattani et al. [81] assessed children’s gestural and language skills at both chronological and corrected age, and they observed that the use of corrected age tended to overestimate the size of children’s gestural repertories in the second year of life. Therefore, we can speculate that considering children’s corrected age in our study may have led to a slight advantage in our group of preterm children. Cattani et al. [81] suggested that employing both corrected and chronological age when studying the gesture and language development of preterm children could provide more detailed information and a clearer view of their developmental processes.

As regards the parental variables examined in the present study, the results indicated a negative effect of higher parental age on the number of gestures and pointing gestures, with children of older parents being more likely to show smaller amounts of such gestures. This finding should be taken with caution, as literature addressing the role of parental age in language delay is very limited. Parental age is certainly a variable that can cover different meanings and aspects of caregiving and child-rearing. Older parents can have more psychological maturity but also more familiar loads, a lack of resources, and higher stress. At the same time, younger parents can be less experienced but have more time and psychological resources to spend with their offspring [53,83,84]. Other minor findings deserve to be mentioned. The first regards the effect of having a family history of language and/or learning disorder; children with a family risk displayed more representational gestures than children without such a risk. This outcome could be interpreted as a strategy to overcome language difficulties in which the gestural representational modality compensates for the lack of verbal labels. A similar pattern has been suggested for children with Down syndrome who have been observed to use more gestures than language-matched children with typical development to compensate for their limitation in the spoken language in the early phases of lexical development [85]. Again, this result should be treated with caution as children with such a family history represented only 18% of the sample. Finally, we observed that children’s cognitive level predicted supplementary gesture–word combinations. This kind of combination is the most complex and mature as it implies the presence of two meaningful elements within a single communicative act; the fact that children with a higher cognitive score produce a higher number of supplementary combinations confirms the strict relationship between cognitive development and the production of representational gestures [70,86].

### 4.1. Limitations, Strengths of the Study and Future Perspectives

Consideration of our findings and their implications must take account of some limitations. A first limitation regards the lack of a longitudinal perspective on our data, which could have offered a more complete insight into the role of gestural communication in the developmental course of language delay. Understanding how the use of gesture and—with specific reference to our outcome—of gesture–word combinations can predict the resolution or persistency of language difficulties is something that needs to be addressed in future studies.

A further limitation worth mentioning regards the book-sharing session. In the first place, this setting was the only observational environment of the study, and, as previously claimed, some of its characteristics could have favored the use of certain kinds of gestures, such as pointing. In the second place, we did not examine the use of gestures of parents during book-sharing, which could have also contributed to individual differences in children’s gestural behaviors. In this respect, the literature showed that mothers of late talkers tend to gesture more than those of typically developing children during a structured task, tuning to late talkers’ scarce lexical abilities [87]. Examining the use of gestures in both members of the interaction would have improved our study design and it could be a perspective for future research.

A third limitation to consider concerns the inclusion of only low-risk preterm children in our sample; it would have been interesting to also investigate the gestural communication of very and extremely preterm children to differentiate their competencies on the basis of their level of immaturity at birth and to make our findings generalizable to the whole preterm population. It should be noted, however, that low-risk preterm children—although representing the majority of the preterm population—are not frequently included in follow-up projects; thus, the present study is particularly relevant in bringing new data about this population.

A further limitation is represented by our sample size. Although the number of participants included in the study is fairly adequate if compared to similar studies retrieved in the literature on this topic [32,33], a wider sample would have disentangled the role of variables that were not much represented in our samples, such as a family history of learning and/or language delay.

Another limit is methodological as short forms of the Italian MB–CDI were filled out by parents about one month before the book-sharing session; associations between observational measures and MB–CDI are therefore not exactly concurrent even if we can suppose a good stability of MB–CDI scores over a one-month interval [88].

A final concern regards the choice of the grammatical measures included in our study. We assessed sentence production, both by computing MLU based on children’s utterances produced during the book-sharing session and with the MB–CDI, but not sentence comprehension. Different studies showed that sentence comprehension is a relevant measure in identifying late talkers [89] and it can be enhanced in late talkers when gestures and speech are integrated into the task [90]. Future studies should address the associations between late talkers’ gesture and gesture–word combination use and sentence comprehension.

On the other hand, our study offers a cross-sectional view on gestural communication in children with language delay assessing gesture production both with direct observation and parental report and examining, as one of the first attempts in literature in this population, the use of gesture-word combination. We believe that accounting for this combinatorial communication form is a noteworthy point of strength of our study. Another relevant aspect of the present study is surely represented by the sample size which is large and homogeneous if compared to previous literature focusing on late talkers’ gestural development, whose age range was often rather wide. To conclude, our study is also the first attempt to investigate the role of individual and environmental variables in accounting for interindividual differences in late talkers’ use of gestures and gesture–word combinations.

### 4.2. Clinical Implications

The study’s main findings offer also relevant insights for assessment and intervention with late talker children. In the first place, these new data on late talkers’ use of communicative gestures, alone or in combination, can be integrated in the assessment of language delay to get a more detailed idea of the points of strength or difficulty for these children. In the second place, a work on the use of gestures and particularly of gesture–word combinations could be embedded in direct (i.e., speech and language therapies, early care family-centered therapies) and indirect (parent or teacher training) interventions with late talkers. Studies have demonstrated that modelling gestures—through direct training or natural exposition to caregivers’ gestural input—can promote children’s language development, especially in children with difficulties in such areas [91,92,93].

## 5. Conclusions

The present study brings new data to enlighten the development of gestures and gesture–word combinations in children with language delay, a topic that has been partially overlooked by literature and that deserves more attention. Overall, late talkers in their second year of life were observed to frequently use communicative gestures during interactions with parent over books, exhibiting the ability to produce gesture–word combinations in their attempt to express multiple semantic elements within an utterance. A consistent use of complementary and, above all, supplementary forms of combinations were associated with higher lexical and grammatical abilities, an association largely documented in children with typical and atypical development, which has been extended for the first time with this study to late talkers. In addition, our findings shed light on how specific individual and environmental variables, which constitute risk factors for late language emergence, also play a role in predicting difficulties in using communicative gestures and gesture–word combinations. Differences in late talkers’ use of gesture and gesture–word combinations were thus associated to several intertwined individual and environmental factors.

## Figures and Tables

**Table 1:** Biological, clinical, and socio-demographic characteristics of late talker children and their parents.

	*M*/*n*	*SD*/%
*Children’s Characteristics*		
Birth status (low-risk preterm), *n*, %	26	42.6
Gestational Age (weeks), Mean, SD	37.04	3.17
Birthweight (grams), Mean, SD	2740.38	843.49
Length of Stay in Hospital (days), Mean, SD	10.72	25.00
Gender (male), *n*, %	41	67.2
Birth order ^a^ (later-born), *n*, %	31	50.8
Twins, *n*, %	15	24.6
Otitis Media, *n*, %	3	4.9
Family History of Language and/or Learning Disorders, *n*, %	11	18.0
Nursery School Attendance, *n*, %	44	72.1
Other Parental Input Besides Italian, *n*, %	8	13.1
Bayley Composite Cognitive Score	88.03	9.67
*Parents’ Characteristics*		
Mother’s Age ^b^ (years), Mean, SD	38.62	5.48
Father’s Age ^c^ (years), Mean, SD	40.87	5.79
Parental Age ^b^ (years), Mean, SD	39.72	5.27
Mothers with High Educational Level (>13 years), *n*, %	38	62.3
Fathers with High Educational Level (>13 years), *n*, %	25	41.0
Mother’s Nationality (Italian), *n*, %	55	90.0
Father’s Nationality (Italian), *n*, %	55	90.0

Note. ^a^ Of the later-borns, 87% were second-borns and 13% were third-borns. ^b^ Missing data *n* = 1. ^c^ Missing data *n* = 8.

**Table 2:** Descriptive statistics of late talkers’ gestures, gesture–word combinations, word tokens, and MLU, during the 10-min parent–child book-sharing interaction.

	*M*	*SD*	Range
Total gestures	36.51	23.03	0–98.84
Pointing gestures	28.63	21.37	0–98.84
Representational gestures	7.89	9.09	0–36.91
Total gesture–word combinations	7.29	8.15	0–33.93
Complementary	5.17	6.66	0–26.17
Equivalent	1.11	2.14	0–12.96
Supplementary	0.99	1.80	0–7.75
Word tokens	22.68	21.81	0–79.94
MLU	1.09	0.12	1–1.42

**Table 3:** Descriptive statistics of late talkers’ gesture/action production and word comprehension (MB–CDI Gestures and Words Short Form) and word and sentence production (MB–CDI Words and Sentences Short Form).

MB–CDI Scores	*M*	*SD*	Range
Gesture/action production	15.18	2.64	7.00–18.00
Word comprehension	87.57	15.56	26.00–100.00
Word production	18.49	12.67	2.00–40.00
Sentence production	2.84	4.00	0.00–12.00

**Table 4:** Pearson’s correlation coefficients among children’s gestural, lexical, and grammatical variables.

	Word Tokens	MLU	Word Comprehension ^a^	Word Production ^a^	Sentence Production ^a^
Total gestures	0.239	0.047	0.172	0.138	0.072
Pointing gestures	0.240	0.101	0.111	0.135	0.097
Representational gestures	0.076	−0.068	0.143	0.030	0.049
Total gesture–word combinations	0.800 **	0.603 **	0.216	0.343 **	0.337 **
Complementary combinations	0.651 **	0.566 **	0.154	0.376 **	0.342 **
Equivalent combinations	0.429 **	0.105	0.017	−0.113	−0.101
Supplementary combinations	0.468 **	0.384 **	0.421 **	0.440 **	0.425 **
Gesture/action production ^b^	0.098	0.022	0.289 *	0.141	0.017

** *p <* 0.01; * *p <* 0.05. **^a^** MB–CDI Words and Sentences Short Form; **^b^** MB–CDI Gesture and Words Short Form.

**Table 5:** Results of multiple linear regression analyses on late talkers’ use of gestures and gesture–word combinations in the book-sharing session and gesture/action production reported at the MB–CDI.

Dependent Variables	Predictors	Standardized β	*t*	*p*	Adj. *R*^2^	*F (df)*	*p*
Total gestures					0.28	6.66 (4, 55)	<0.001
	Birth status (low-risk preterm)	0.29	2.52	0.015			
	Gender (male)	−0.26	−2.31	0.025			
	Birth order (later-born)	0.27	2.42	0.019			
	Parental age	−0.46	−3.95	<0.001			
Pointing gestures					0.19	5.76 (3, 56)	0.002
	Birth status (low-risk preterm)	0.29	2.42	0.019			
	Birth order (later-born)	0.27	2.31	0.024			
	Parental age	−0.41	−3.40	0.001			
Representational gestures					0.07	5.65 (1, 58)	0.021
	Family history of LLD	0.30	2.38	0.021			
Total Gesture–word Combinations					0.11	8.46 (1, 58)	0.005
	Birth status (low-risk preterm)	0.36	2.91	0.005			
Complementary Combinations					0.13	5.54 (2, 57)	0.006
	Birth status (low-risk preterm)	0.30	2.49	0.016			
	Gender (male)	−0.28	2.32	0.024			
Equivalent Combinations	None						
Supplementary Combinations					0.17	6.88 (2, 57)	0.002
	Birth status (low-risk preterm)	0.30	2.51	0.015			
	Cognitive score	0.35	2.90	0.005			
Gestures/action production ^					0.07	5.56 (1, 58)	0.022
	Gender (male)	−0.30	2.36	0.022			

^ MB–CDI Gesture and Words Short Form.

## Data Availability

The dataset presented in this article is not readily available because it includes sensitive information about minors with developmental vulnerabilities. Requests to access the dataset should be directed to corresponding authors.

## Appendix A

**Table A1:** Full-term and low-risk preterm participants’ biological, clinical, and socio-demographic characteristics.

	Full-Term Children (*n =* 35)	Low-Risk Preterm Children (*n* = 26)	χ^2^/t	*df*	*p*
Gestational Age (weeks), Mean (SD)	39.37 (1.28)	33.90 (2.02)	12.95	59	<0.001
Birthweight (grams), Mean (SD)	3347.26 (464.00)	1923 (452.78)	11.97	59	<0.001
Length of Stay in Hospital (days), Mean (SD)	2.63 (1.52)	21.61 (35.80)	−2.70	59	0.003
Gender (male), *n* (%)	23 (65.7)	18 (69.2)	0.08	1, 61	0.772
Birth order (later-born), *n* (%)	16 (45.7)	14 (53.8)	0.39	1, 61	0.530
Twins, *n* (%)	0 (0.0)	15 (57.7)	26.78	1, 61	<0.001
Otitis Media, *n* (%)	2 (5.7)	1 (3.8)	0.11	1, 61	0.739
Family History of Language and/or Learning Disorders (LLD), *n* (%)	6 (17.1)	5 (19.2)	0.04	1, 61	0.834
Nursery School Attendance, *n* (%)	27 (77.1)	17 (65.4)	1.03	1, 61	0.311
Other Parent Input Besides Italian, *n* (%)	4 (11.4)	4 (15.4)	0.17	1, 60	0.683
Bayley Composite Cognitve Score	88.43 (10.06)	87.50 (9.30)	−0.87	59	0.380
Caesarean Section, *n* (%)	8 (22.9)	24 (92.3)	28.85	1, 61	<0.001
SGA, *n* (%)	1 (2.9)	5 (19.2)	4.51	1, 61	0.034
RDS, *n* (%)	0 (0.0)	14 (53.8)	24.46	1, 61	<0.001
Apnea, *n* (%)	0 (0.0)	1 (3.8)	1.37	1, 61	0.242
MV, *n* (%)	0 (0.0)	2 (7.7)	2.78	1, 61	0.095
BDP, *n* (%)	0 (0.0)	1 (3.8)	1.37	1, 61	0.242
Clinical or culture-proven sepsis, *n* (%)	0 (0.0)	1 (3.8)	1.37	1, 61	0.242
Hyperbilirubinemia requiring Phototherapy, *n* (%)	3 (8.6)	9 (34.6)	21.85	1, 61	<0.001
IVH any grade, *n* (%)	0 (0.0)	0 (0.0)	-	-	-
ROP any stage, *n* (%)	0 (0.0)	0 (0.0)	-	-	-
Mother’s Age (years), Mean (SD) ^a^	37.26 (5.33)	40.38 (5.26)	−2.26	58	0.028
Father’s Age (years), Mean (SD) ^b^	39.41 (5.72)	42.63 (5.49)	−2.07	51	0.043
Parental Age (years), Mean (SD) ^a^	38.48 (5.15)	41.34 (5.07)	−2.15	58	0.036
Mothers with High Educational Level (>13 years), *n* (%)	25 (71.4)	14 (53.8)	2.00	1, 61	0.157
Fathers with High Educational Level (>13 years), *n* (%)	18 (51.4)	7 (26.9)	3.71	1, 61	0.540
Mother’s Nationality (Italian), *n* (%)	32 (91.4)	23 (88.5)	0.15	1, 61	0.700
Father’s Nationality (Italian), *n* (%)	32 (91.4)	23 (88.5)	0.15	1, 61	0.700

SGA: Small for gestational age, infants with a birthweight <10th percentile for gestational age; RDS: respiratory distress syndrome; MV: mechanical ventilation; BDP: bronchopulmonary dysplasia; IVH: intra-ventricular hemorrhage; ROP: retinopathy of prematurity. Note. ^a^ Missing data *n* = 1. ^b^ Missing data *n* = 8.

**Table A2:** Correlations between children’s gestural measures and individual and environmental factors.

	Birth Status (Low-Risk Preterm)	Gender (Male)	Birth Order (Later-born)	Family History of LLD	Cognitive Score	Chronological Age	Maternal Age	Paternal Age	Maternal Education	Paternal Education
Total gestures	0.150	−0.263 *	0.264 *	0.154	0.121	0.093	−0.305 *	−0.381 **	−0.092	−0.07
Pointing gestures	0.176	−0.214	0.237	0.044	0.063	0.069	−0.283 *	−0.333 *	−0.142	−0.107
Representational gestures	0.016	−0.200	0.149	0.304 *	0.232	0.07	−0.172	−0.142	0.042	0.103
Total combinations	0.368 **	−0.214	0.068	0.121	0.155	0.307 *	−0.034	−0.008	−0.100	−0.021
Complementary combinations	0.216	0.056	−0.036	0.217	0.042	0.173	−0.123	0.071	−0.078	−0.003
Equivalent combinations	0.301 *	−0.276 *	0.087	−0.068	0.132	0.269 *	0.010	−0.023	−0.053	0.011
Supplementary combinations	0.283 *	−0.192	−0.128	0.027	0.336 **	0.097	−0.010	−0.023	−0.019	−0.178
Gestures/action production ^	−0.071	−0.298 *	0.054	0.105	−0.008	0.087	−0.204	−0.130	0.064	−0.041

** *p <* 0.01; * *p <* 0.05. ^ MB–CDI Gesture and Words Short Form.
